# The UAP2-BRR2b Module Regulates Low-Temperature Hypocotyl Elongation by Modulating Pre-mRNA Splicing in *Arabidopsis*

**DOI:** 10.3390/ijms27209111

**Published:** 2026-10-14

**Authors:** Xinwei Zhao, Feiyang Chen, Ziyi Hu, Leping Geng, Jianhua Zhu

**Affiliations:** 1 School of Life Sciences, Anhui Agricultural University, Hefei 230036, China; zhao2023036@ahau.edu.cn (X.Z.);; 2 Research Center for Biological Breeding, Advanced Academy, Anhui Agricultural University, Hefei 230036, China

**Keywords:** *Arabidopsis thaliana*, cold stress, pre-mRNA splicing, alternative splicing

## Abstract

Plants adapt to low temperatures through transcriptomic reprogramming, yet how the core spliceosome maintains splicing fidelity during cold-regulated development remains poorly understood. Here, we investigated the role of the U4/U6.U5 tri-snRNP-associated protein 2 (AtUAP2) in *Arabidopsis* using genetic, transcriptomic, and biochemical approaches. Under normal conditions (22 °C), *uap2*, *brr2b*, and *uap2 brr2b* mutants showed no obvious developmental defects. Upon low-temperature treatment, however, *uap2* displayed a severe impairment in cold-dependent hypocotyl elongation. RNA-sequencing revealed that UAP2 deficiency induced widespread pre-mRNA mis-splicing under cold stress. Ectopic expression of correctly spliced *AtGRP7* and *AtLOS2* cDNAs in *uap2* partially restored hypocotyl growth under cold conditions, supporting their roles as key downstream effectors. Mechanistically, protein interaction assays demonstrated that UAP2 physically binds to the U5 RNA helicase BRR2b. While *brr2b* mutants exhibited a hypocotyl growth phenotype similar to *uap2*, the *uap2 brr2b* double mutant displayed an enhanced growth defect under low temperatures. Overall, our findings suggest that the UAP2-BRR2b complex operates as a functional module required to sustain spliceosomal efficiency under low temperature, providing insights into the potential connection between core splicing machinery dynamics and cold-induced hypocotyl growth responses in *Arabidopsis*.

## 1. Introduction

As sessile organisms, plants have evolved sophisticated regulatory mechanisms to withstand fluctuating environmental conditions [1,2]. Abiotic stresses, such as extreme temperatures, drought, and salinity, significantly limit plant growth and global crop productivity [3]. To survive these challenges, plants execute a rapid and precise reprogramming of their transcriptome. While transcriptional control has long been the focus of stress research, it is now evident that post-transcriptional processes, particularly those ensuring the fidelity of mRNA maturation, provide a critical layer of regulation that allows plants to maintain cellular homeostasis under adverse environments [4,5,6].

Among abiotic constraints, cold stress is a primary factor affecting plant survival and geographical distribution [7]. In *Arabidopsis thaliana*, cold acclimation is fundamentally governed by the CBF/DREB1-dependent signaling pathway, which functions as a central regulatory hub [8,9]. The induction of *CBF* genes is precisely controlled by upstream master regulators, most notably the transcription factor ICE1 [10]. The activity of ICE1 is further fine-tuned through post-translational modifications, involving the E3 ligase HOS1 for degradation and the protein kinase OST1 for stabilization [11]. Moreover, studies have highlighted the importance of chromatin remodeling in this process; for instance, HOS15 associates with histone deacetylases to modulate the epigenetic status of *CBF* promoters, ensuring a rapid transcriptional response [12,13]. These signaling pathways lead to high levels of *Cold-Responsive* (*COR*) gene expression. However, low temperature increases the thermodynamic stability of inhibitory RNA secondary structures, which can impede the molecular machinery responsible for gene expression. Consequently, the maintenance of efficient RNA processing—specifically pre-mRNA splicing—has emerged as a vital “checkpoint” for cold tolerance, ensuring that the transcripts of essential genes are correctly matured and translated [14,15].

Pre-mRNA splicing is executed by the spliceosome, a dynamic macromolecular machine composed of five nuclear ribonucleoproteins (snRNPs) [16,17]. The splicing cycle requires a series of highly orchestrated rearrangements, moving from the pre-catalytic *B* complex to the activated B* complex [18,19]. A pivotal step in this transition is the formation and subsequent activation of the U4/U6.U5 tri-snRNP [20,21]. Within the pre-catalytic tri-snRNP module, U4/U6.U5 tri-snRNP-associated proteins serve as structural core components that not only help maintain complex integrity but also regulate the RNA helicase BRR2; this regulatory interaction ensures precise control of BRR2’s U4/U6 duplex unwinding activity, which is the essential molecular switch for spliceosome activation [21,22,23]. Recent studies have indicated that core splicing factors, such as STA1, are involved in environmental responses [24,25,26]; however, the coordination between U4/U6.U5 tri-snRNP-associated proteins and BRR2 in facilitating these structural transitions remains poorly understood. Given the increased rigidity of RNA duplexes at low temperatures, the regulatory interaction within the tri-snRNP module—where the scaffolding protein structurally organizes and restrains the RNA helicase BRR2—is hypothesized to be critical for ensuring the precise and timely unwinding of the U4/U6 duplex, thereby maintaining spliceosome activation kinetics under cold stress.

In this study, we show that UAP2 regulates cold-dependent hypocotyl elongation in *Arabidopsis*. Transcriptome analysis revealed widespread splicing defects in *uap2* under low temperature, which are partially restored by expressing correctly spliced *AtGRP7* or *AtLOS2*. Among these targets, AtGRP7 is a cold-inducible glycine-rich RNA-binding protein that acts as a post-transcriptional regulator and RNA chaperone during cold adaptation, whereas AtLOS2 is a bifunctional enolase/transcriptional repressor essential for cold-responsive gene expression and low-temperature growth. Furthermore, UAP2 interacts with BRR2b to form a functional module that maintains spliceosomal efficiency, providing potential insights into how core pre-mRNA splicing may coordinate with low-temperature hypocotyl growth responses.

## 2. Results

### 2.1. AtUAP2 Is Required for Cold-Dependent Hypocotyl Elongation in Arabidopsis

**Figure 1:**
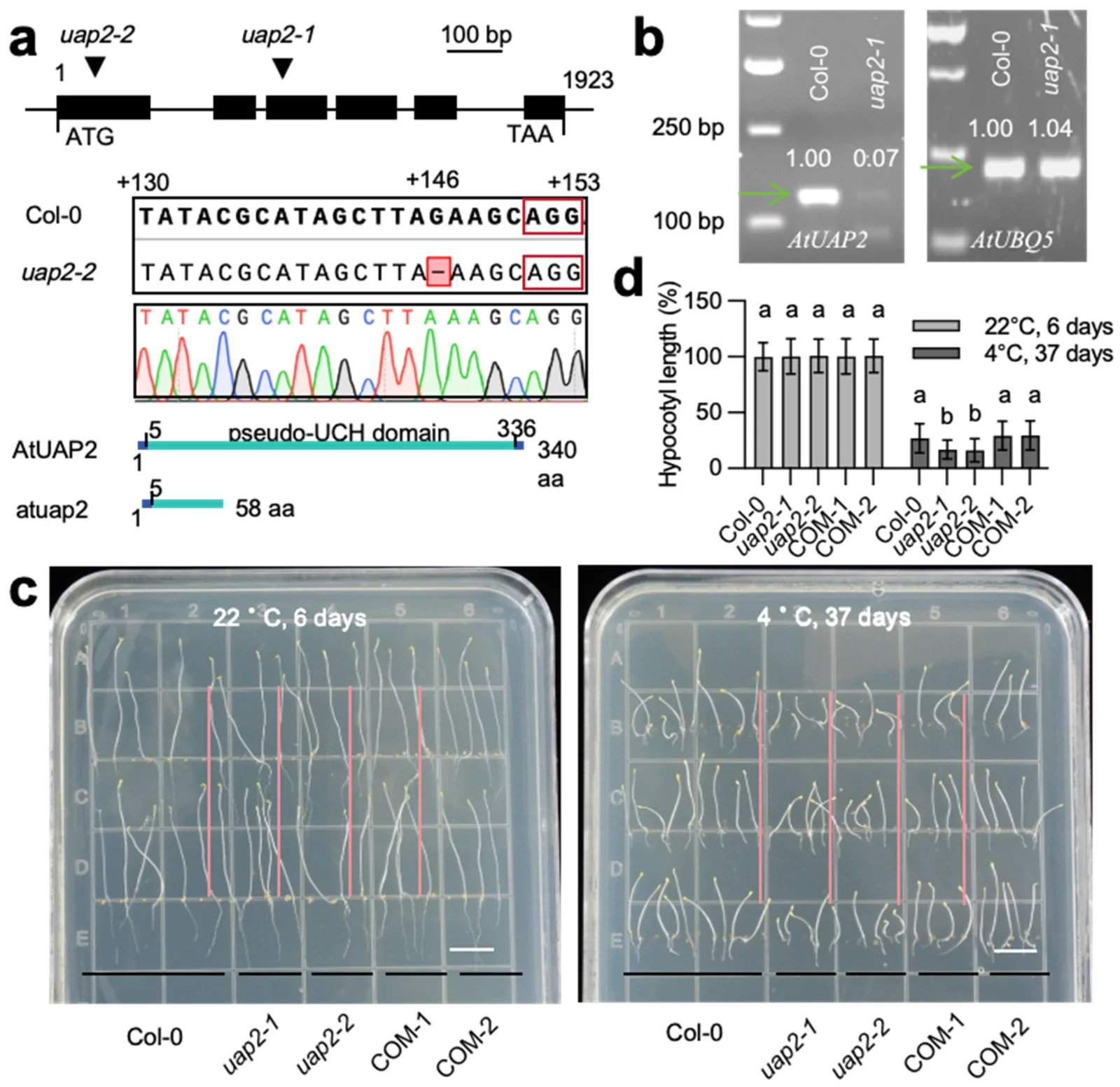
Molecular characterization of AtUAP2 and phenotypes of *uap2* mutants under cold stress. (**a**) Schematic representation of the *AtUAP2* gene structure and mutation sites. Exons and introns are indicated by black boxes and horizontal lines, respectively. Arrowheads denote the T-DNA insertion site in *uap2-1* (SALK_125945) and the gRNA target site for CRISPR/Cas9-mediated editing in *uap2-2*. The gRNA target site is shown with the protospacer adjacent motif (PAM, ‘AGG’) outlined in a red box. The sequencing chromatogram confirms a single-nucleotide deletion of ‘G’ at position +146 bp in the *uap2-2* line. Predicted protein structures are shown for the full-length AtUAP2 (340 aa) and the truncated protein in *uap2-2* (58 aa). AtUAP2 contains a pseudo-UCH domain (a catalytically inactive ubiquitin C-terminal hydrolase-like domain). (**b**) Semi-quantitative RT-PCR analysis of *UAP2* transcript levels in Col-0 and *uap2-1* seedlings. *AtUBQ5* was used as an internal loading control. Total RNA was reverse-transcribed, and PCR was performed for 32 cycles for *UAP2* and 26 cycles for *UBQ5*. Equal volumes (5 μL per lane) of the PCR products were loaded into agarose gels. The relative expression level of *UAP2* in *uap2-1* was calculated after normalizing to *UBQ5*. (**c**) Representative phenotypes of Col-0, *uap2-1*, *uap2-2*, and two independent complementation lines (COM-1 and COM-2) grown under control conditions (22 °C in the dark for 6 days) and cold stress (4 °C in the dark for 37 days). Scale bars = 1 cm. (**d**) Relative hypocotyl length of the indicated genotypes at 4 °C relative to 22 °C (%). Data are presented as means ± SD (n = 100). Different lowercase letters above the bars indicate significant differences determined by Two-way ANOVA followed by Tukey’s multiple comparisons test at *p* < 0.05.

To identify regulators of low-temperature growth, we screened an *Arabidopsis* T-DNA insertion population from the ABRC (stock CS27941), comprising 6866 sequence-genotyped loci. Through a forward genetic screen focusing on low-temperature seedling growth and hypocotyl elongation, we isolated several mutants displaying impaired cold-dependent growth. Among them, SALK_125945 carries a T-DNA insertion within the third exon of At4g22410, a gene encoding the U4/U6.U5 tri-snRNP-associated protein 2 (AtUAP2). Consequently, this mutant allele was named *uap2-1* (Figure 1a). RT-qPCR and gel densitometric analysis confirmed that the UAP2 transcript level was drastically reduced to 0.07 in *uap2-1* compared with Col-0 (1.00), demonstrating a severe loss-of-function allele (Figure 1b). To rule out potential genetic background effects or second-site mutations in *uap2-1*, we employed the CRISPR/Cas9 system to generate an independent knockout allele, *uap2-2* (Figure 1a). This line contains a single-nucleotide deletion in the first exon, introducing a premature stop codon that results in a severely truncated protein of 58 amino acids (aa) compared to the 340 aa wild type (Figure 1a). We also generated two independent complementation lines (COM-1 and COM-2) by expressing the native-promoter-driven *UAP2* genomic sequence in the *uap2-1* background.

To evaluate low-temperature hypocotyl growth, seedlings were grown on MS medium supplemented with 2% sucrose under continuous darkness. We monitored etiolated hypocotyl elongation, a developmental process sensitive to temperature changes. Under the control condition (22 °C for 6 days), *uap2-1*, *uap2-2*, and both complementation lines displayed no significant differences in hypocotyl elongation compared to Col-0 (Figure 1c,d). However, under cold conditions (4 °C for 37 days), hypocotyl elongation was severely inhibited in both *uap2* mutants, whereas the COM lines successfully restored wild-type hypocotyl length (Figure 1c,d).

To test whether UAP2 is involved in other abiotic stress responses, we examined mutant performance under salinity and heat conditions. Under salt stress (100 mM NaCl), both *uap2* mutants displayed relative root lengths and fresh weights indistinguishable from Col-0 (Figure S1a,c). Similarly, in a dark-induced hypocotyl elongation assay following a transient heat shock (45 °C for 70 min), relative growth inhibition remained comparable across all genotypes, with no statistically significant differences between the wild type and *uap2* mutants (Figure S1d,e). Together, these results indicate that UAP2 is not required for salt-inhibited root growth or heat-induced hypocotyl suppression, highlighting its relatively selective role in low-temperature hypocotyl growth responses.

### 2.2. Genome-Wide Splicing Analysis Identifies UAP2 as a Key Regulator of Pre-mRNA Splicing Under Low Temperature

Although *AtUAP2* expression remained relatively stable in Col-0 seedlings throughout a 24-h cold treatment, unlike the rapidly induced *AtCBF1* (Figure S2a), we performed global RNA-seq to profile UAP2-dependent splicing dynamics. We chose the 24-h cold treatment timepoint to specifically target persistent, phenotype-driving mis-splicing events during low-temperature responses, rather than early, transient fluctuations. To capture transcriptome dynamics, we harvested 12-day-old seedlings of Col-0 and both *uap2* alleles grown under control conditions or subjected to cold treatment (4 °C for 24 h). Prior to performing global RNA-seq, we extracted RNA from these collected samples and evaluated the expression of the cold-responsive marker gene *AtCOR15a* via RT-qPCR. The results showed a robust and significant upregulation of *AtCOR15a* across all genotypes, validating that the low-temperature treatment was highly effective (Figure S2b). Having confirmed the success of the cold treatment, we immediately proceeded with high-throughput RNA sequencing.

**Figure 2:**
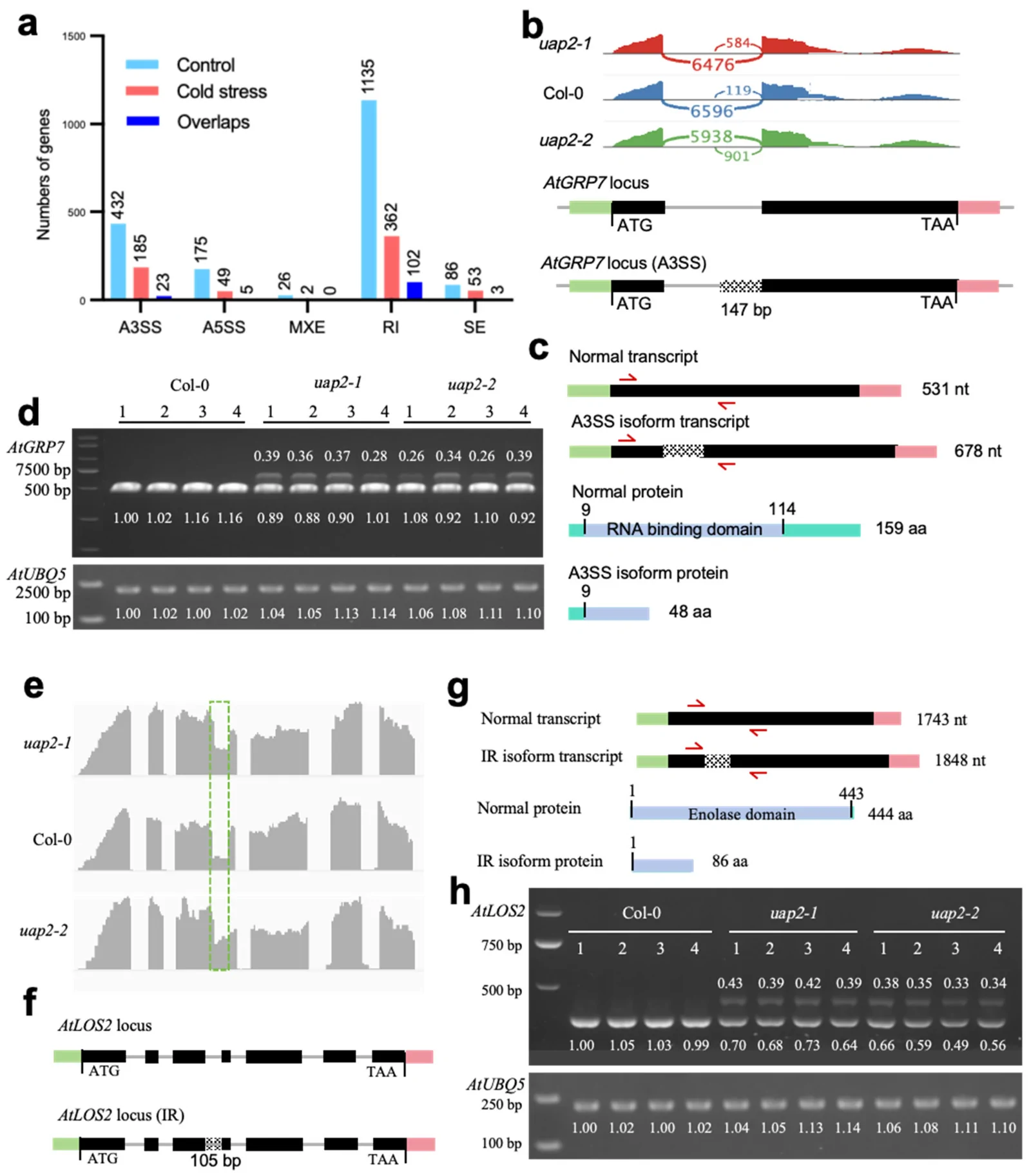
Analysis of global alternative splicing and splicing fidelity in *uap2* mutants. (**a**) Classification and quantification of differential alternative splicing (DAS) genes in *uap2* mutants compared to Col-0 in control condition (22 °C), cold stress condition (4 °C for 24 h) and overlaps of both conditions. DAS categories include alternative 3′ splice site (A3SS), alternative 5′ splice site (A5SS), mutually exclusive exons (MXE), retained intron (RI), and skipped exon (SE). (**b**) Sashimi plot of shifted alternative 3′ splice site (A3SS) at the *AtGRP7* locus in *uap2* mutants and Col-0 under cold stress. The sashimi plots represent the merged signal from four independent biological replicates per line. The peaks represent exonic regions, while arcs connect splice junctions. Numerical labels on the arcs indicate the count of reads supporting each specific splice event. Below: schematic illustration of the *AtGRP7* locus that would undergo normal or alternative splicing (A3SS, 147bp). (**c**) Schematic representation of the *AtGRP7* splicing patterns and the resulting protein products. The normal transcript encodes a full-length protein (159 aa) containing an RNA-binding domain (blue). The A3SS isoform transcript introduces a premature termination codon, resulting in a severely truncated protein (48 aa). (**d**) Semi-quantitative RT-PCR validation of *AtGRP7* A3SS in Col-0 and *uap2* mutants under cold stress. The upper and lower bands correspond to the A3SS isoform and normal transcript, respectively. Numbers on the agarose gels indicate the measured DNA band intensities of splicing isoforms. *AtUBQ5* was used as an internal reference. (**e**) IGV coverage tracks showing the read distribution at *AtLOS2* locus in Col-0 and *uap2* mutants under cold stress. The green dashed box highlights the retention of the third intron (105 bp). The IGV coverage tracks represent the merged signal from four independent biological replicates per line. (**f**) Schematic illustration of the *AtLOS2* locus that would undergo normal splicing or retained intron (IR). (**g**) Schematic representation of the *AtLOS2* splicing patterns and the resulting protein products. The normal transcript encodes a full-length protein (444 aa) containing an enolase domain (green). The IR isoform transcript introduces a premature termination codon, resulting in a severely truncated protein (86 aa). (**h**) Semi-quantitative RT-PCR validation of *AtLOS2* splicing patterns in Col-0 and *uap2* mutants. The upper and lower bands correspond to the IR isoform and normal transcript, respectively. Numbers indicated on the agarose gels represent the measured DNA band intensities, reflecting the differences in splicing pattern between genotypes. *AtUBQ5* was used as an internal control.

Bioinformatic analysis revealed that UAP2 is essential for maintaining splicing fidelity (NCBI GEO accession no. GSE346033). Using a False Discovery Rate (FDR) ≤ 0.05 and an absolute inclusion level difference (|*IncLevelDifference*|) > 0.02 as thresholds for significant differential splicing, we identified 1,854 and 651 differentially alternatively spliced (DAS) genes in *uap2* mutants compared to Col-0 under control and cold conditions, respectively (Figure 2a, Tables S2–S11). Among the various DAS categories, retained intron (RI) emerged as the most predominant type of aberrant splicing in *uap2*, followed by alternative 3’ splice sites (A3SS) (Figure 2a). Across both growth conditions, 133 genes were differentially spliced in *uap2* mutants compared to Col-0 (Figure 2a, Table S12). Interestingly, only 49 genes were significantly differentially expressed in *uap2* mutants compared to Col-0 under cold stress, none of which were known key regulators of low-temperature growth (Table S13). This suggests that the hypocotyl growth defects in *uap2* are primarily driven by post-transcriptional splicing defects rather than overall transcriptional reprogramming.

### 2.3. UAP2 Maintains the Splicing Fidelity of AtGRP7 and AtLOS2 Under Cold Stress

Among the DAS genes identified in the *uap2* transcriptome, we specifically focused on *AtGRP7* (Glycine-Rich Protein 7) and *AtLOS2* (ENOLASE 2), two known positive regulators of low-temperature growth and cold responses. Systematic screening of our DAS datasets revealed that major canonical cold-signaling and hypocotyl-elongation genes (e.g., *CBF1/2/3*, *HY5*, and *PIF4*) did not exhibit detectable alternative splicing changes. This could be attributed to the fact that our RNA-seq was performed using whole-seedling bulk tissues; thus, tissue- or cell-type-specific DAS events occurring in localized regions (such as the hypocotyl elongation zone) might be diluted below detection thresholds, leaving *AtGRP7* and *AtLOS2* as the primary high-confidence, robustly detectable target genes in our bulk dataset.

Sashimi plot analysis of the *AtGRP7* locus under cold stress revealed a significant shift of an A3SS in *uap2* mutants, resulting in an enrichment of a 147-bp sequence inclusion (Table S7, Figure 2b). While the normally spliced transcript yields a full-length 159-amino acid (aa) protein, this cold-induced A3SS isoform introduces a premature termination codon (PTC), predicted to produce a severely truncated 48-aa product (Figure 2c). Semi-quantitative RT-PCR validated these RNA-seq findings, showing an enrichment of the upper band (the 147-bp inclusion isoform) in both *uap2* alleles under cold treatment (Figure 2d).

In addition to *AtGRP7*, bioinformatic analysis identified *AtLOS2* as another critical direct target of UAP2. The RNA-seq dataset revealed that intron retention (RI) within *AtLOS2* transcripts was significantly exacerbated in *uap2* mutants under cold stress (Table S10). Integrated Genomics Viewer (IGV) visualization confirmed a marked accumulation of the third intron of *AtLOS2* in the mutants (Figure 2e,f). This RI event introduces a PTC, generating a non-functional truncated protein of 86 aa (Figure 2g). The splicing defect was substantiated by semi-quantitative RT-PCR; while the correctly spliced transcript predominated in Col-0, both *uap2* alleles exhibited a consistent and reproducible increase in the RI isoform across four biological replicates (Figure 2h).

### 2.4. Expression of AtGRP7 and AtLOS2 Partially Restores Low-Temperature Hypocotyl Growth in uap2

**Figure 3:**
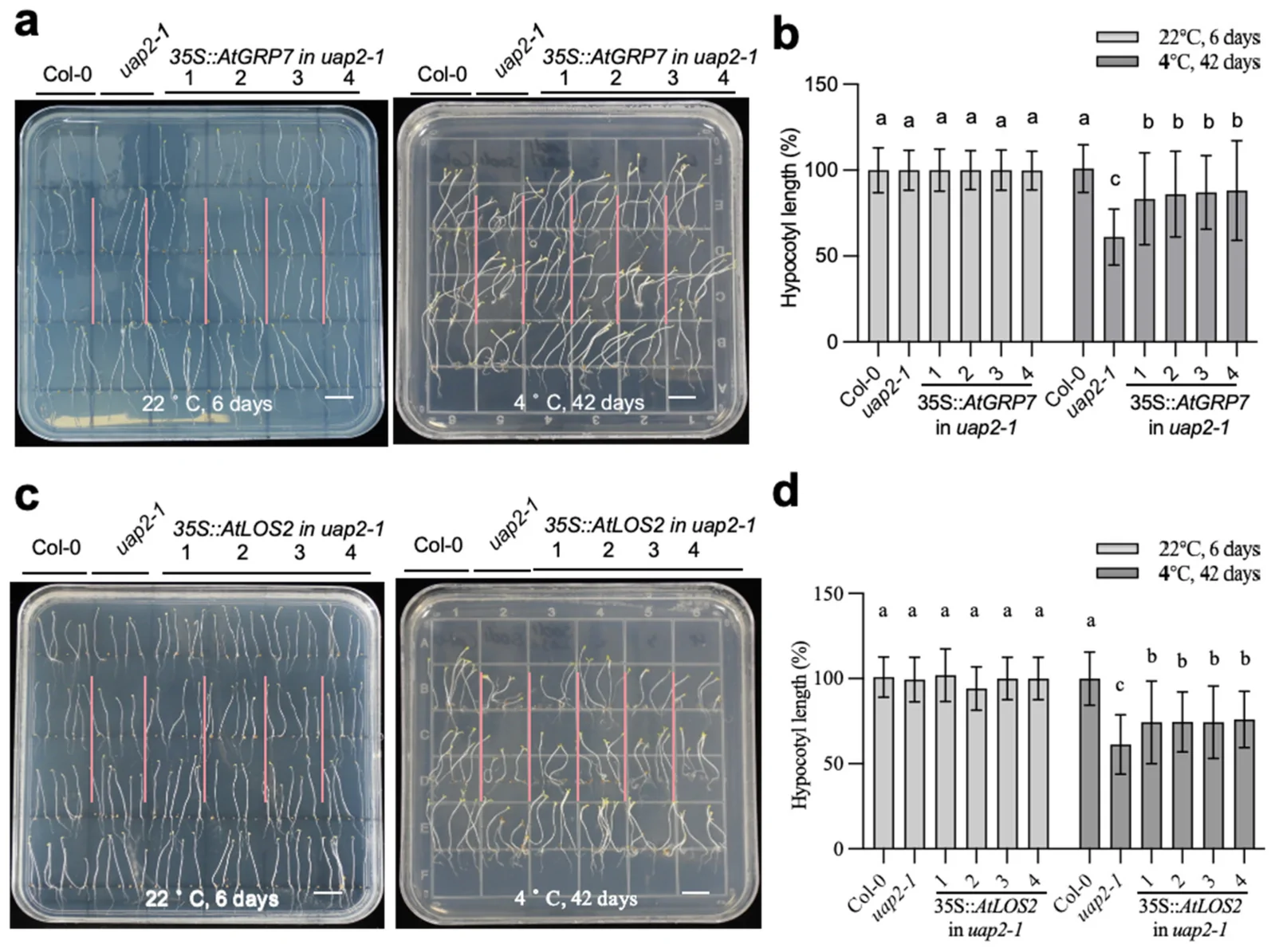
Genetic complementation of *uap2* cold-sensitive phenotypes using *AtGRP7* or *AtLOS2*. (**a**,**c**) Representative images of Col-0, *uap2-1*, and four *AtGRP7* (**a**) or *AtLOS2* (**c**) transgenic lines grown at 22 °C in the dark (6-day) and at 4 °C in the dark (42-day). Scale bars = 1 cm. (**b**,**d**) Relative hypocotyl length of the *AtGRP7* (**b**) or *AtLOS2* (**d**) genotypes at 4 °C relative to 22 °C (%). Data are presented as means ± SD (n = 100). Different lowercase letters above the bars indicate significant differences determined by Two-way ANOVA followed by Tukey’s multiple comparisons test at *p* < 0.05.

To investigate the functional significance of *AtGRP7* and *AtLOS2* mis-splicing, we generated transgenic lines expressing their respective full-length coding sequences (CDS) under the control of the 35S promoter in the *uap2-1* background. These independent lines exhibited varying transcript accumulation levels for *AtGRP7* (1.1- to 10.0-fold) and *AtLOS2* (0.8- to 6.1-fold) relative to Col-0 (Figure S2e,f). Under control conditions (22 °C for 6 days), no significant differences in hypocotyl elongation were observed among genotypes (Figure 3a–d). Under cold treatment (4 °C for 42 days), however, the introduction of correctly spliced *AtGRP7* or *AtLOS2* led to a significant, albeit partial, restoration of hypocotyl elongation compared with the *uap2-1* mutant (Figure 3a–d). Taken together, these genetic data demonstrate that providing functional AtGRP7 or AtLOS2 proteins can compensate for splicing defects, confirming both genes as downstream effectors required for UAP2-mediated low-temperature hypocotyl growth.

### 2.5. UAP2 Physically Interacts with the N-Terminal Domain of BRR2b

**Figure 4:**
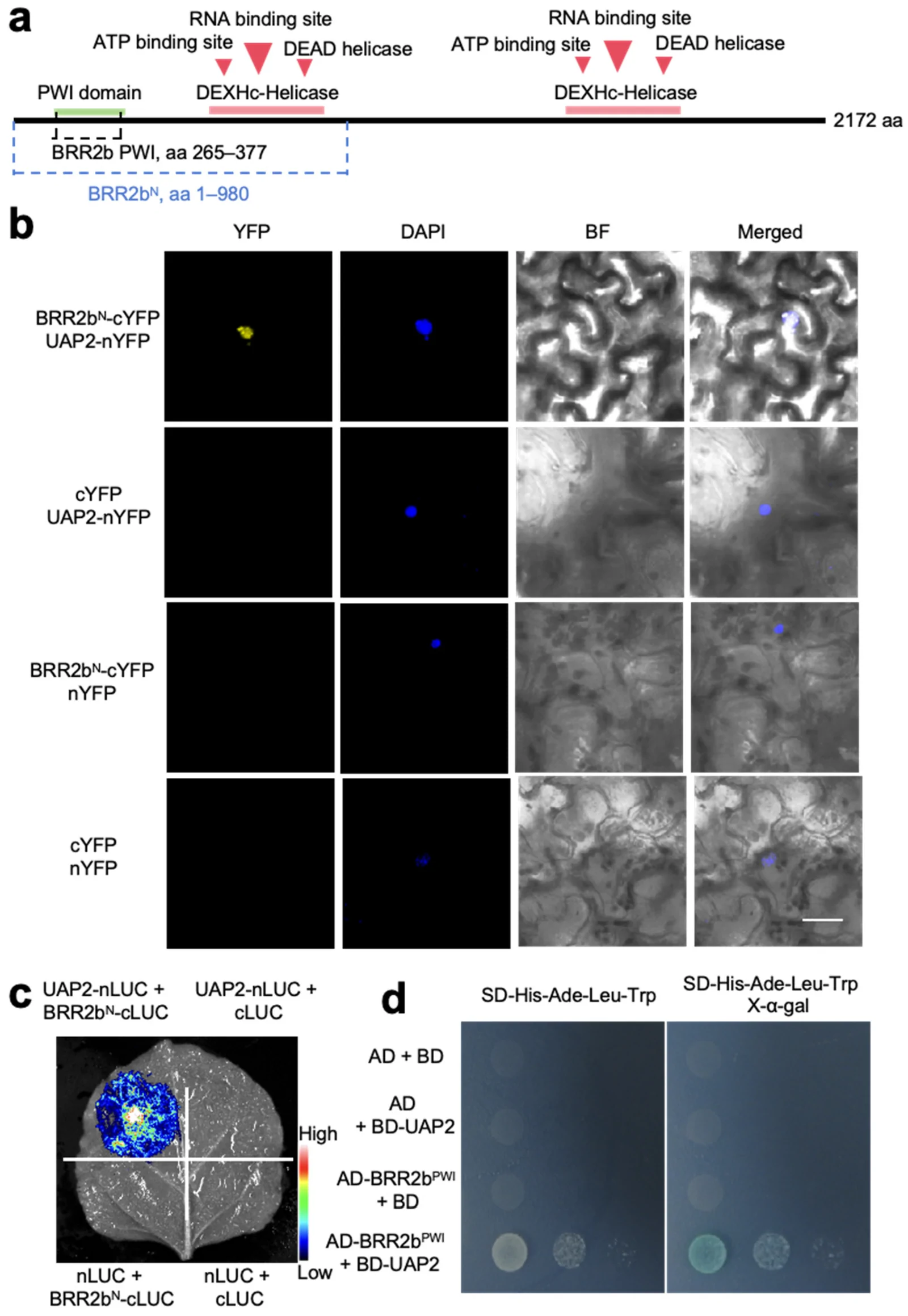
Analysis of the physical interaction between UAP2 and BRR2b proteins. (**a**) Schematic representation of the BRR2b protein. The 2172-aa protein contains an N-terminal PWI domain and tandem DEXHc-Helicases. The N-terminal region (BRR2bN, residues 1–980) and the specific PWI domain (residues 265–377) within this region are highlighted. These two fragments were utilized in subsequent assays to define the binding interface with UAP2. (**b**) Luciferase Complementation Imaging (LCI) assay demonstrating the interaction of UAP2 and BRR2bN (aa 1–980). Bar = 50 μm. (**c**) Bimolecular Fluorescence Complementation (BiFC) assay confirming the interaction between UAP2 and the BRR2b-N region (BRR2bN, aa 1–980) in *N. benthamiana* leaves. (**d**) Yeast two-hybrid (Y2H) assays verifying the interaction between UAP2 and BRR2b PWI domain.

To elucidate the UAP2 molecular mechanism, it is critical to identify the protein partners that cooperate with the UAP2 within the spliceosome. Initial in silico analysis using the STRING database (https://string-db.org) predicted a high probability of physical association between UAP2 and BRR2b (*AT2G42270*), one of the two *Arabidopsis* BRR2 paralogs (Figure S3a). Remarkably, this physical association between the UAP2 and BRR2 homologs is evolutionarily conserved from yeast to humans, highlighting its fundamental role within the spliceosome [19,23,27,28]. While STRING also predicted BRR2a, we focused on BRR2b because it positively regulates cold responses [29] and verified that *brr2b* mutants were readily available. The 2172-aa protein of BRR2b contains an N-terminal PWI domain and tandem DEXHc-Helicases (Figure 4a). To gain structural insights into this predicted association, we modeled the UAP2–BRR2b complex using AlphaFold3. This analysis identified a potential interaction interface involving a network of residues (Figure S3b). This potential interaction is of particular interest given that the transition from the pre-catalytic *B* complex to the activated spliceosome (*B^act^*) represents a pivotal regulatory checkpoint, which strictly depends on the U5-specific RNA helicase BRR2 to unwind the U4/U6 duplex [30,31]. These observations prompted us to systematically characterize the physical association between UAP2 and BRR2b through a suite of complementary biochemical assays.

To verify the predicted physical association between UAP2 and BRR2b in vivo, we first performed Bimolecular Fluorescence Complementation (BiFC) and firefly luciferase complementation imaging (LCI) assays in *Nicotiana benthamiana* leaves. Given that full-length BRR2b is a large protein (2173 amino acids) and AlphaFold3 modeling predicted that its UAP2-binding interface resides within the N-terminal region, the N-terminal fragment of BRR2b (BRR2^N^, encoding the N-terminal fragment, residues 1–980) was utilized for these interaction assays. Co-expression of UAP2-nYFP and BRR2b^N^-cYFP yielded robust YFP signal specifically in the nuclei (Figure 4b). Consistent with these findings, the LCI assay showed strong luminescence signals in leaf areas co-transformed with UAP2-nLUC and BRR2b^N^-cLUC (Figure 4c). To further delineate the interaction domain, we employed the yeast two-hybrid (Y2H) system using different BRR2b fragments. While UAP2 failed to interact with BRR2b^N^ (Figure S3c)—likely due to improper heterologous folding in yeast—it exhibited a robust interaction with the shorter, isolated PWI domain (residues 265–377) (Figure 4d).

### 2.6. UAP2 and BRR2b Act Synergistically as a Dedicated Functional Module to Regulate Low-Temperature Hypocotyl Growth

**Figure 5:**
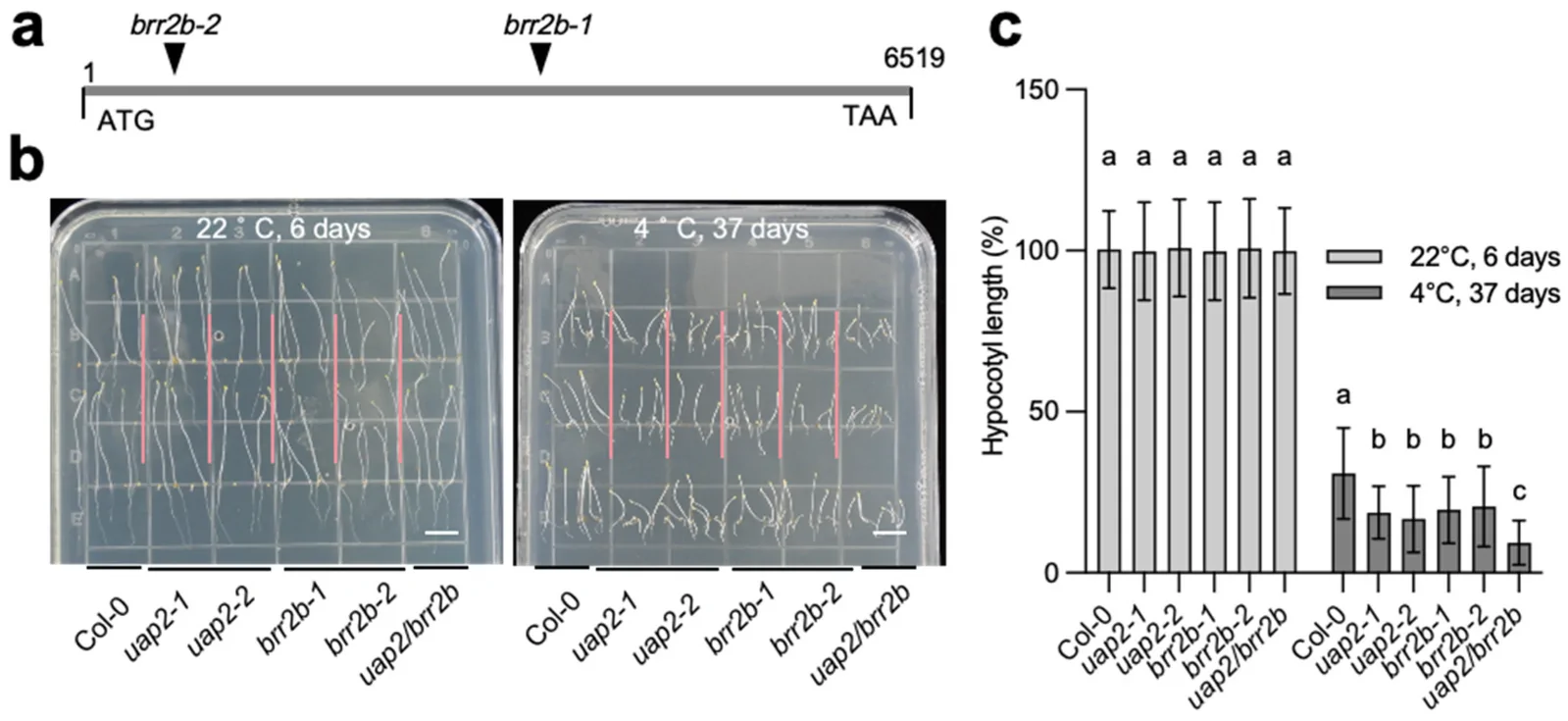
Genetic interaction between UAP2 and BRR2b. (**a**) Schematic representation of the *AtBRR2b* gene structure. Red triangles indicate the T-DNA insertion sites for two independent alleles: *brr2b-1* (SALK_034237) and *brr2b-2* (SALK_048780). Note that the BRR2b coding sequence (6519 bp) is intronless. (b, c) Representative phenotypes (**b**) and relative hypocotyl length (**c**) of Col-0, *uap2*, brr2b, and *uap2 brr2b* mutants grown under control (22 °C, 6 days) and cold stress (4 °C, 37 days) conditions in dark. The Y-axis in (**c**) represents the percentage of hypocotyl length at 4∘C relative to that at 22 °C (%). Data are presented as means ± SD (n = 100). Different lowercase letters above the bars indicate significant differences determined by Two-way ANOVA followed by Tukey’s multiple comparisons test at *p* < 0.05. Scale bars = 1 cm.

To further elucidate the genetic hierarchy between UAP2 and BRR2b in low-temperature growth responses, we identified two independent T-DNA insertion alleles for BRR2b: *brr2b-1* (SALK_034237) and *brr2b-2* (SALK_048780) (Figure 5a). Additionally, the homozygous *uap2-1 brr2b-1* double mutant was generated through genetic crosses for subsequent phenotypic analysis.

**Figure 6:**
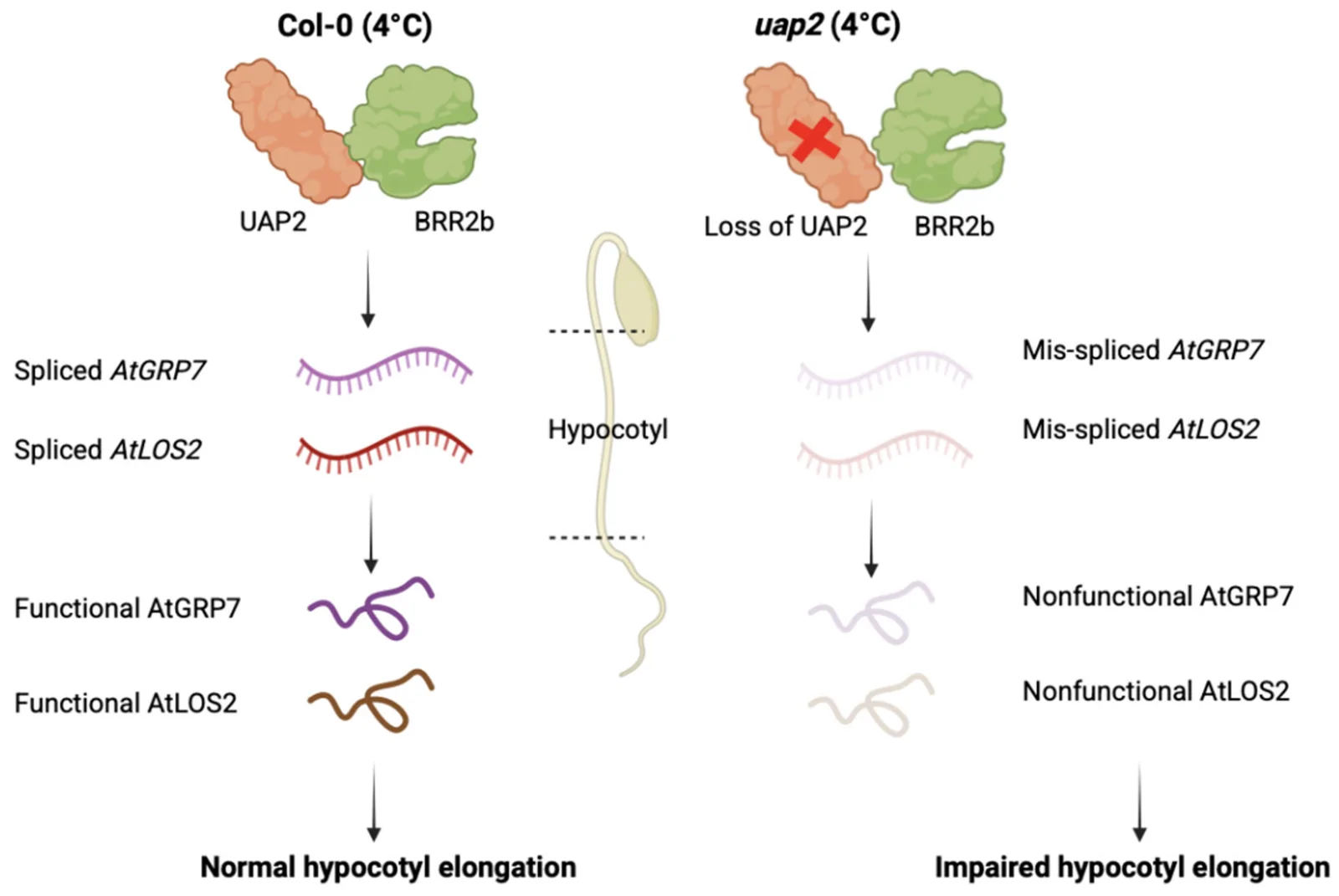
Proposed working model for the UAP2-BRR2b module in regulating low-temperature hypocotyl elongation. Under low-temperature conditions (4 °C), UAP2 interacts with BRR2b to ensure correct pre-mRNA splicing of *AtGRP7* and *AtLOS2*, thereby promoting functional protein accumulation and hypocotyl elongation (**left**). Loss of UAP2 leads to mis-splicing of these downstream targets and impaired hypocotyl growth under cold stress (**right**).

Low-temperature hypocotyl growth of these genetic lines was evaluated using the dark-induced hypocotyl elongation assay. Under control conditions (22 °C), neither the single mutants nor the *uap2-1 brr2b-1* double mutant exhibited any developmental anomalies or significant differences in hypocotyl length compared to Col-0 (Figure 5b,c). This pivotal negative result rules out the possibility that these splicing factors simply exert a constitutive, housekeeping effect on general plant growth. Under cold treatment (4 °C), however, hypocotyl elongation was significantly compromised in all mutants. While both *uap2* and *brr2b* single mutants displayed comparable, pronounced defects in hypocotyl elongation, the *uap2-1 brr2b-1* double mutant exhibited an even more severe, synergistic reduction in hypocotyl length (Figure 5b,c). Based on these findings, we propose a working model outlining how the UAP2-BRR2b module coordinates pre-mRNA splicing to sustain low-temperature hypocotyl growth (Figure 6).

## 3. Discussion

Pre-mRNA splicing catalyzed by the spliceosome, a highly dynamic macromolecular machine, is a critical post-transcriptional regulatory node in plant adaptation to environmental anomalies [5,32,33]. Emerging evidence indicates that rapid transcriptomic reprogramming under abiotic stress relies heavily on the precise coordination and structural remodeling of core spliceosomal components to maintain global splicing fidelity [6,29]. However, how these non-stress-induced core splicing factors dynamically sustain their catalytic efficiency under specific restrictive conditions, such as low temperature, remains poorly understood. Our findings provide key insights into this mechanism by demonstrating that while the *uap2* mutant displays a low-temperature hypocotyl growth defect (Figure 1 and Figure S1), *AtUAP2* expression itself is not induced by cold treatment (Figure S2a). This suggests that UAP2 may not operate primarily as a cold-induced transcriptional “responder,” but rather serves as a core component of the splicing machinery whose functional requirement becomes particularly critical during low-temperature growth responses.

Relative to Col-0, the number of differentially alternatively spliced (DAS) genes in *uap2* mutants was substantially higher under control conditions (22 °C, 1854 genes) than under cold treatment (4 °C for 24 h, 651 genes). At first glance, if UAP2 operates as a key regulator in low-temperature growth, a larger cohort of cold-induced DAS events might be expected. This pattern can be explained by two complementary factors. Biologically, low temperature triggers a general reduction in cellular metabolic activity and transcriptional flux, effectively shrinking the total pool of pre-mRNA substrates available for splicing [34]. Methodologically, this decline may also reflect our selected 24-h timepoint. While a transient, widespread surge of mis-splicing events typically occurs during the immediate early phase of cold shock [35], a 24-h exposure captures the mid-to-late phase of low-temperature responses, during which the plant has initiated homeostatic adjustments and transcriptional deceleration. Therefore, the 651 DAS genes identified at 24 h likely represent persistent downstream targets that rely on UAP2 to sustain splicing fidelity, rather than an exhaustive catalog of all cold-responsive transcripts. Gene Ontology (GO) biological process analysis further revealed that these DAS genes under 22 °C and 4 °C are mainly involved in key metabolic and stress-response pathways (Figure S2c,d).

Through DAS analysis and semi-quantitative RT-PCR validation, we confirmed that *AtGRP7* undergoes A3SS selection, while *AtLOS2* exhibits IR in the *uap2* mutants under cold stress (Tables S7 and S10, Figure 2). *AtGRP7* encodes a pivotal cold-induced RNA-binding protein that functions as an RNA chaperone, binding mRNAs to melt inhibitory secondary structures and ensure transcript stability and translation under low temperatures [36,37,38]. *AtLOS2* encodes a bifunctional enolase traditionally known to regulate the canonical *CBF-COR* transcriptional cascade via *ZAT10* repression [39,40]; however, our transcriptomic data showed no widespread failure of the canonical *CBF-COR* cascade in *uap2* (Table S13). Introducing correctly spliced cDNAs of either *AtGRP7* or *AtLOS2* partially restored low-temperature hypocotyl growth in the *uap2* background (Figure 3). These genetic complementation lines indicate that the impaired low-temperature hypocotyl elongation in *uap2* is, at least in part, causally driven by the concurrent splicing failures of these downstream regulators. Notably, given the tissue-specific expression patterns of *AtGRP7* and *AtLOS2*, our use of bulk RNA-sequencing might dilute cell-type-specific transcriptional changes [39,41]. This methodological limitation could explain the subtle overall expression shifts despite the severe splicing and developmental phenotypes observed.

Previous studies have shown that *AtGRP7* and *AtLOS2* are markedly upregulated under cold stress [36,37,39]. This robust induction may create a “transcriptional burden,” increasing the reliance of these transcripts on the UAP2–BRR2b module for efficient splicing processing. While their elevated expression levels may drive their mis-splicing in the *uap2* mutant, whether these targets possess distinct sequence features—such as suboptimal splice sites or complex secondary structures—that differentiate them from genes that remain correctly spliced under low temperature remains to be further investigated. Beyond *AtGRP7* and *AtLOS2*, the widespread alternative splicing defects identified in *uap2* mutants suggest a complex post-transcriptional regulatory network (Tables S2–S11). Our DAS dataset serves as a valuable genomic resource for future identification of novel genes that rely on this splicing coordination to support low-temperature morphogenetic responses. While our current study establishes the essential role of UAP2 in low-temperature hypocotyl elongation, whether this splicing mechanism similarly governs other cold-regulated developmental responses across different tissue types remains an interesting subject for future investigation.

Our data establish the UAP2-BRR2b module as a coordinated functional unit essential for pre-mRNA splicing efficiency. It is worth noting that while the BRR2b^N^ construct interacted with UAP2 in BiFC and LCI assays, this interaction was not detected in Y2H. Such discrepancies are common, as Y2H systems often yield false negatives due to missing plant-specific modifications or co-factors [42]. Crucially, UAP2 directly interacted with the isolated BRR2b PWI domain in Y2H (Figure 4d), confirming their intrinsic physical association. While direct biochemical evidence of UAP2 modulating BRR2b’s RNA helicase activity remains to be fully elucidated, insights from yeast and human orthologs provide a solid foundation for our model [23,27]. In yeast, the UAP2 ortholog Sad1 directly counteracts Brr2-mediated dissociation of the U4/U6.U5 tri-snRNP to maintain spliceosomal homeostasis. This evolutionary conservation strongly suggests that *Arabidopsis* UAP2 similarly coordinates with BRR2b to stabilize the tri-snRNP complex and sustain splicing fidelity under low-temperature conditions [27]. The enhanced defects in low-temperature hypocotyl elongation observed in the *uap2 brr2b* double mutant (Figure 5b,c) suggest a breakdown of genetic buffering. *Arabidopsis* possesses multiple paralogs, including two for *UAP2* (*AT4G22350* and *AT4G22285*) and one for *BRR2b* (BRR2a, *AT1G20960*). In single mutants, basal splicing fidelity may be partially maintained by these functional paralogs; however, the simultaneous loss of both *UAP2* and its primary partner *BRR2b* likely causes a severe disruption in spliceosome activation that surpasses the compensatory capacity of the remaining homologs. It should be noted that, due to the large size of full-length BRR2b (2172 aa) and technical challenges associated with its yield and stability in recombinant expression systems, our in vivo interaction analyses (Y2H, BiFC, and LCI) were performed using the N-terminal domain of BRR2b, which was predicted by AlphaFold3. However, the computational modeling cannot fully account for the dynamic conformational transitions, native stoichiometry, or full-length structural constraints of BRR2b in vivo. Thus, validating the interaction using full-length BRR2b via Co-IP or full-length reporter systems remains an important objective for future biochemical studies.

Based on our biochemical and genetic findings, we propose a working model for the UAP2–BRR2b module under cold stress (Figure 6). Under low-temperature conditions (4 °C), physical interaction between UAP2 and BRR2b maintains spliceosomal functionality, ensuring proper pre-mRNA splicing of target downstream genes, including *AtGRP7* and *AtLOS2*. This accurate splicing event promotes the production of functional effector proteins required to sustain low-temperature hypocotyl elongation. Conversely, in the *uap2* mutant, disruption of this spliceosomal module impairs splicing fidelity, accumulating mis-spliced transcripts and nonfunctional proteins, which ultimately results in impaired cold-induced hypocotyl growth. While our RNA-seq analysis has identified UAP2-dependent splicing targets, a global transcriptomic profile and alternative splicing analysis of the *brr2b* mutant under low temperature are currently unavailable. Future comparative studies mapping the alternative splicing landscapes in both *uap2* and *brr2b* will be essential to definitively delineate their shared downstream targets. Nevertheless, the striking phenotypic similarity under low temperature and the synergistic interaction observed in the double mutant strongly support our model that these two proteins function as a unified pathway to facilitate cold-dependent growth responses.

## 4. Materials and Methods

### 4.1. Plant Materials and Growth Conditions

The *Arabidopsis thaliana* T-DNA insertion lines utilized for the forward genetic screen were obtained from the Arabidopsis Biological Resource Center (ABRC, stock number CS27941). This sequence-genotyped population comprises 6866 independent loci generated by the Salk Institute. Among these lines, the *uap2-1* mutant (SALK_125945) carries a T-DNA insertion within the third exon of *AtUAP2* (*At4g22410*). Homozygous *uap2-1* mutant plants were identified and confirmed by PCR genotyping using gene-specific and T-DNA border primers. For the screening of chilling-sensitive regulators, seeds were surface-sterilized and plated on Murashige and Skoog (MS) medium supplemented with 2% (*w*/*v*) sucrose and solidified with 1.2% (*w*/*v*) agar. To reliably score low-temperature seedling growth and etiolated hypocotyl elongation, plates were kept in the dark at 4 °C. *Arabidopsis thaliana* ecotype Columbia-0 (Col-0) was used as the wild-type. The T-DNA insertion mutants *brr2b-1* (SALK_034237) and *brr2b-2* (SALK_048780) were obtained from the ABRC. The *uap2-2* mutant was generated by the CRISPR-Cas9 system. The *uap2/brr2b* mutant was generated by crossing *uap2-1* with *brr2b-1* and confirmed by genotyping in the F_2_ generation, and F_3_ generation was used for subsequent analyses.

For *Arabidopsis* growth, seeds were surface-sterilized, stratified at 4 °C for 2 days in the dark, and sown on Murashige and Skoog (MS) medium supplemented with 3% (*w*/*v*) sucrose and 0.8% (*w*/*v*) agar. Plants were grown under long-day conditions (16-h light/8-h dark) at 22 °C with a light intensity of 120 μmol m^−2^ s^−1^. For soil growth, seedlings were transferred to a mixture of peat moss and vermiculite (3:1) after 10 days and grown under the same conditions.

### 4.2. Mutant Generation and Genotyping

The *UAP2* (*AT4G22410*) single-guide RNA (sgRNA) was cloned into the p0056 vector. The construct was transformed into *Agrobacterium tumefaciens* strain GV3101 and introduced into Col-0 plants via the floral dip method [43]. T_1_ transgenic plants were selected on hygromycin, and the target site was sequenced to identify mutations. A homozygous line carrying a 1-bp deletion (*uap2-2*) was isolated in the T_2_ generation, and the T_3_ generation was used for subsequent analyses. Genomic DNA was extracted using a CTAB-based method [44]. Homozygous T-DNA insertion mutants were identified using a combination of gene-specific and T-DNA border primers (see Table S1).

### 4.3. Stress Phenotype Assays

Seeds were surface-sterilized and sown on MS medium supplemented with 2% (*w*/*v*) sucrose solidified with 1.2% (*w*/*v*) agar. After stratification at 4 °C in the dark for 2 days, the plates were placed vertically under continuous light at 22 °C for 18 h to ensure synchronized germination.

#### 4.3.1. Cold Stress Assay

Following germination synchronization, seedlings were divided into two groups. For the control group, plates were maintained vertically at 22 °C in darkness for 6 days. For the cold treatment group, plates were incubated vertically at 4 °C in darkness for 37–42 days. Hypocotyl length was quantified using ImageJ software (Version: 2.14.0/1.54f). For each genotype and treatment, every 100 seedlings from three independent biological replicates were measured.

#### 4.3.2. Salt Stress Assay

Four-day-old Col-0 and mutant seedlings were transferred to MS medium (1.2% agar) supplemented with or without 100 mM NaCl. The initial position of the primary root tip was marked on the back of the plate. After 8 days of vertical growth at 22 °C, the total root elongation was recorded. Root length was measured using ImageJ software (Version: 2.14.0/1.54f), with 20 seedlings analyzed per treatment across three biological replicates. For fresh weight determination, seedlings were harvested 8 days after transfer. Excess moisture was gently blotted from the plants using filter paper, and the fresh weight was immediately measured using an analytical balance. For each genotype and treatment, the total fresh weight of 20 seedlings per replicate was recorded, with three independent biological replicates performed. Data were presented as means ± SD, and statistical significance was evaluated using Student’s *t*-test.

#### 4.3.3. Heat Stress Assay

Seedlings were subjected to heat treatment at 45 °C for 70 min, while control plates remained at 22 °C. Following treatment, all plates were transferred to a 22 °C growth chamber in the dark. Hypocotyl length was measured and photographed 6 days post-treatment. Data were collected from 100 seedlings per genotype, and the experiment was repeated three times independently.

### 4.4. RNA Extraction, RT-PCR, and Quantitative RT-PCR (RT-qPCR)

Total RNA was extracted from whole seedlings using the RNAprep Pure Plant Kit (TIANGEN, Beijing, China). Gene-specific primers (Table S1) were manually designed and aligned against the Arabidopsis thaliana genome to confirm their unique sequence specificity. Prior to quantitative analysis, standard semi-quantitative RT-PCR followed by 1.5% agarose gel electrophoresis was performed to verify that each primer pair produced a single, specific amplification product of the expected size without non-specific bands or primer-dimers.

All RT-qPCR primers were evaluated using the Vazyme Tm Calculator (https://crm.vazyme.com/cetool/tmcal.html) for T_m_ optimization (58–62 °C) and NCBI Primer-BLAST against the *Arabidopsis thaliana* genome to verify target specificity and exclude potential secondary structures or primer-dimers. For RT-qPCR, reactions were performed in triplicate using a SYBR Green master mix on a real-time PCR system (CFX96; Bio-Rad Laboratories, Hercules, CA, USA). The thermal cycling conditions were as follows: initial denaturation at 95 °C for 30 s, followed by 40 cycles of 95 °C for 5 s and 60 °C for 30 s. Relative expression levels were calculated using the 2^−ΔΔCt^ method. RT-qPCR reaction specificity was validated by melting curve analysis (60 to 95 °C), ensuring a single specific dissociation peak for each product, and subsequently confirmed by agarose gel electrophoresis.

### 4.5. RNA Sequencing and Splicing Analysis

Total RNA was extracted from 12-day-old Col-0 and *uap2* seedlings under control (22 °C) and cold-stress (4 °C for 24 h) conditions. Four biological replicates per genotype and treatment were used. RNA-seq libraries were constructed and sequenced on an Illumina platform to generate 150-bp paired-end reads. Raw reads were processed for quality control using FastQC (v0.10.1) and quality-trimmed using Cutadapt (v1.9.1) to remove adapter sequences and low-quality bases (Phred score < 20). The clean reads were aligned to the *Arabidopsis thaliana* reference genome using Hisat2 (v2.2.1). Gene expression levels were quantified as FPKM using Htseq (v0.6.1), and differential expression analysis was performed with DESeq2 (v1.26.0). Alternative splicing (AS) events were identified and quantified using rMATS (v4.1.0). Differentially alternatively spliced (DAS) genes were defined by a false discovery rate (FDR) ≤ 0.05 and an absolute inclusion level difference (IncLevel Difference) > 0.02. Gene Ontology (GO) enrichment analysis was conducted using the GOseq package (v1.38.0), utilizing the Wallenius non-central hyper-geometric distribution to account for gene length bias.

### 4.6. LCI, BiFC and Y2H Assay

The coding sequences UAP2 and the BRR2b N-terminal fragment (aa 1-980) were fused to the N-terminal (nLUC) and C-terminal (cLUC) halves of firefly luciferase in pCAMBIA1300 vectors, respectively. The constructs were transformed into *Agrobacterium tumefaciens* strain GV3101. Recombinant bacterial cultures were harvested, washed, and resuspended in the infiltration buffer (10 mM MES, pH 5.6, 10 mM MgCl_2_, and 150 μM AS) to an OD_600_ of 0.6–0.8. After incubation at room temperature in darkness for 2–3 h, the suspensions were co-infiltrated into fully expanded leaves of 4- to 5-week-old *N. benthamiana* plants. Luminescence signals were detected 48 h later using a low-light cooled CCD imaging system (ChemiDoc MP Imaging System; Bio-Rad Laboratories, Hercules, CA, USA) after spraying with 1 mM luciferin.

The coding sequences of UAP2 and the BRR2b N-terminal fragment (aa 1-980) were fused to the N-terminal (nYFP) and C-terminal (cYFP) halves of YFP, respectively, in pCAMBIA1300-35S-MCS-HA-n/cYFP-35S-Hyg vectors. Constructs were co-transfected into *Agrobacterium* strain GV3101 and infiltrated into leaves of 4-week-old *Nicotiana benthamiana*. YFP fluorescence was observed 48 h post-infiltration using a confocal laser scanning microscope (Zeiss LSM 880; Carl Zeiss AG, Oberkochen, Germany). The YFP signals were excited using a 514 nm argon laser, and emission was captured in the 520–550 nm range.

The coding sequences of UAP2 and the PWI domain of BRR2b were cloned into the pGBKT7 (BD) and pGADT7 (AD) vectors, respectively. The constructs were co-transformed into yeast strain Y2HGold. Transformants were selected on SD/-Trp/-Leu medium and subsequently spotted onto SD/-Trp/-Leu/-His/-Ade medium. Interactions were assessed by growth after 4 days at 30 °C. The α-Galactosidase activity was assayed using X-α-Gal as a substrate.

It should be noted that heterologous positive controls (standard known interacting protein pairs) were not assayed in all Y2H, BiFC, and LCI assays to benchmark absolute system sensitivity.

### 4.7. Complementation and Overexpression Assays

The genomic fragment of *UAP2* (including 2262-bp native promoter) was cloned into the pCAMBIA1305 vector and transformed into the *uap2-1* mutant to generate complementation lines (COM). For rescue experiments, the coding sequences (CDS) of correctly spliced *AtLOS2* (*AT2G36530*) and *AtGRP7* (*AT2G21660*) were driven by the CaMV 35S promoter and cloned into the pCAMBIA1300 vector. These constructs were transformed into the *uap2-1* mutant. Homozygous T_3_ lines were used for phenotypic analysis under cold stress.

### 4.8. AlphaFold3 Structural Prediction and Molecular Visualization

Structural models for the UAP2–BRR2b complex (using both full-length BRR2b) were predicted using the AlphaFold3 server under default settings with standard seed initialization. Model confidence was evaluated using Predicted Local Distance Difference Test (pLDDT) and Predicted Aligned Error (PAE) scores. Structural alignments, spatial distance measurements, and polar interactions were analyzed using PyMOL (v2.5). Hydrogen bonds between UAP2 and BRR2b interface residues were identified and visualized with a distance threshold of 3.2 Å.

### 4.9. Statistical Analysis

All experiments were performed with at least three independent biological replicates. Statistical significance was determined using GraphPad Prism 9. Data significance among multiple groups was determined using Two-way ANOVA followed by Tukey’s multiple comparisons test. Means labeled with different letters represent statistically significant differences at *p* < 0.05.

## Data Availability

The data presented in this study are openly available in the NCBI Gene Expression Omnibus (GEO) database and are accessible through GEO Series accession number GSE346033. at https://www.ncbi.nlm.nih.gov/geo/ [GSE346033].

## Supplementary Materials

The following supporting information can be downloaded at: https://www.mdpi.com/article/10.3390/ijms27209111/s1.
